# Longitudinal Assessment of Intersegmental Abnormalities in the Lumbar Spine of Adolescent Competitive Alpine Skiers Over 48 Months

**DOI:** 10.1177/03635465241295384

**Published:** 2025-01-01

**Authors:** Georg C. Feuerriegel, Daniela Meyer, Daniel P. Fitze, Jonas Hanimann, Christoph Stern, Flavia Schürmann, Stefan Fröhlich, Johannes Scherr, Jörg Spörri, Reto Sutter

**Affiliations:** † Department of Radiology, Balgrist University Hospital, Faculty of Medicine, University of Zurich, Zurich, Switzerland; ‡ Sports Medical Research Group, Department of Orthopaedics, Balgrist University Hospital, University of Zurich, Zurich, Switzerland; § University Centre for Prevention and Sports Medicine, Department of Orthopaedics, Balgrist University Hospital, University of Zurich, Zurich, Switzerland; Investigation performed at Balgrist University Hospital, University of Zurich, Zurich, Switzerland

**Keywords:** overuse injuries, athletes, skiing, low back pain

## Abstract

**Background:**

Overuse-related intersegmental abnormalities in the spine of competitive alpine skiers are common findings. However, longitudinal changes in intersegmental abnormalities and symptoms throughout adolescence have not been assessed.

**Purpose:**

To longitudinally assess and compare overuse-related spinal intersegmental abnormalities in adolescent competitive alpine skiers over 48 months and to compare magnetic resonance imaging (MRI) findings in asymptomatic and symptomatic skiers.

**Study Design:**

Case series; Level of evidence, 4.

**Methods:**

Adolescent competitive alpine skiers were prospectively recruited between November 2108 and February 2019 and underwent 3-T MRI of the lumbar spine at baseline and after 48 months. All MRI scans were assessed for intersegmental changes of the intervertebral disk, vertebral body, and facet joints. At both time points, athletes’ low back pain (LBP) symptoms were assessed via retrospective interviews relating to the 12-month period before the MRI study. Athletes were classified as symptomatic if at least 1 substantial episode of health problems related to back overuse had occurred in the 12 months before the MRI examination. The Wilcoxon signed-rank test and Pearson chi-square test were used to compare the measurements.

**Results:**

A total of 63 athletes (mean age at follow-up, 19.6 ± 1.2 years; 25 female) were included in the study. A significant increase in LBP affecting training and competition was observed at follow-up, with only 2 athletes reporting a history of spinal trauma (baseline, n = 13; follow-up, n = 20; *P* = .04). Of the athletes with LBP (n = 27), 59% (n = 16) reported recurrent LBP, 15% (n = 4) reported permanent LBP, and 26% (n = 7) reported 1-time LBP since baseline. Assessment of intersegmental changes revealed a significant increase in the number of athletes with disk signal reduction (baseline, n = 10; follow-up, n = 21; *P* = .001), disk bulging (baseline, n = 7; follow-up, n = 19; *P* = .002), or disk herniation (follow-up, n = 2; *P* = .04). Overall, intersegmental abnormalities did not correlate with LBP within the last year (*P* = .53).

**Conclusion:**

Overuse-related intersegmental abnormalities of the lumbar spine are common in adolescent competitive alpine skiers and are often clinically silent at this age. These abnormalities may persist throughout skeletal maturation and even worsen during adolescence.

In athletes, the prevalence of low back pain (LBP) is strongly dependent on the type of sport, but it has been reported to range from 18% to 65% and is a common cause of absence from training and competition.^25,26^ LBP in professional athletes, in contrast to the general population, is often associated with overuse injuries due to high training volumes, periods of increased load, and years of exposure, characterized by gradual onset, recurrent symptoms, progression over time, and lack of identifiable precipitating events.^9,15,27^ This is supported by the fact that athletes often have more intersegmental abnormalities than nonathletes. A previous study showed a higher incidence of spondylolysis in adolescent athletes of various disciplines compared with nonathletes (32% vs 2%).^16^ Similarly, a study by Witwit et al^28^ found significantly more segmental abnormalities such as disk herniation and reduced disk height in adolescent soccer players compared with nonathletes (89% vs 54%).

In competitive alpine skiing, the back has been recognized as a common area of overuse injury in adolescent and young adult athletes.^3,11^ This can be explained by the loading patterns of alpine skiing and related off-snow training, which place unfavorable excessive loads on the lumbar spine. During off-snow training, an accumulation of heavy loads acting on the spine, especially if the recovery time between loads is not adequate, can lead to adverse tissue stress and structural deterioration.^17^ During alpine skiing, the continuous combination of high vibration loads, frontal bending, lateral bending, and torsion in the loaded trunk is associated with high disk loading and likely leads to a high prevalence of overuse-related LBP.^19-21^ Given that there is no identifiable event after which overuse-related LBP develops, early recognition and appropriate prevention are key to avoiding injuries that could lead to reduced athletic performance or, in the worst case, early retirement from competitive sports.

The standard for assessing early structural changes in the lumbar spine is magnetic resonance imaging (MRI).^1,29^ High-resolution imaging allows the assessment of subtle changes in intervertebral disks, vertebral endplates, nerve roots, and facet joints.^7,22^ A higher rate of spinal abnormalities detected using MRI and radiography has previously been reported in youth and adolescent alpine and mogul skiers than in an age-matched control cohort.^2,8,15,24^ The mogul skiers had significantly more structural abnormalities such as disk degeneration and endplate changes (mean, 7.25) compared with the controls (mean, 3.78; *P* < .023).^24^ It has also been reported that the presence of lumbar spine abnormalities is associated with a greater risk of LBP later in an athlete's career, suggesting that early detection and appropriate prevention and treatment are essential.^12-14^ However, studies assessing the changes in overuse-related intersegmental abnormalities of the lumbar spine over a longer period of time, particularly during the adolescent and young adult phases of an athlete's life, are scarce. Knowing which changes are self-limiting or persist throughout skeletal maturation could help us to gain a better understanding of LBP in competitive alpine skiers and develop effective prevention measures.

Therefore, the aims of this study were (1) to longitudinally assess and compare overuse-related intersegmental abnormalities of the spine using 3-T MRI in youth competitive alpine skiers over 48 months and (2) to compare the MRI findings of asymptomatic and symptomatic skiers and to explore their clinical relevance at the 4-year follow-up.

## Methods

### Participant Selection

**Figure 1.**
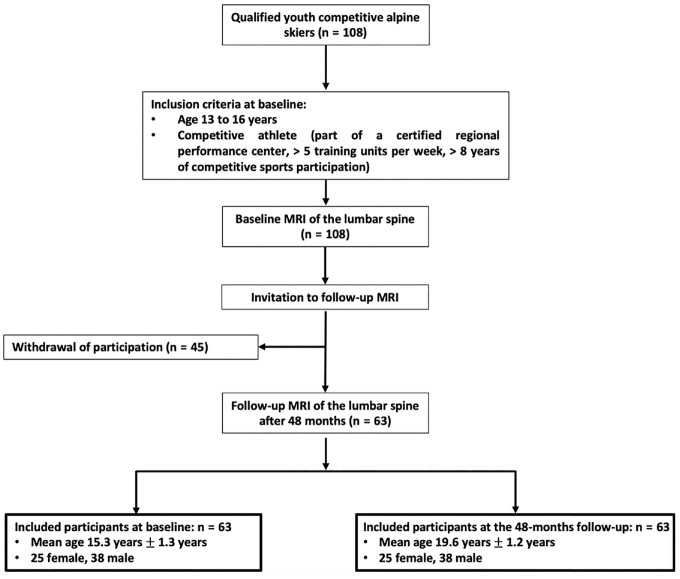
Patient selection flowchart. In all participants, 3-T magnetic resonance imaging (MRI) of the lumbar spine was performed at baseline and at the 48-month follow-up.

Youth competitive alpine skiers were prospectively recruited between November 2018 and February 2019 for a cross-sectional MRI investigation (hereinafter called “baseline”). To be considered a qualified youth competitive skier, participants had to be part of an accredited regional performance center with more than 5 training units per week and have participated in competitive alpine sports for more than 8 consecutive years (n = 108) (Figure 1, Table 1). All participants underwent 3-T MRI of the lumbar spine at baseline. In total, 63 participants were rescanned after 48 months (hereinafter called “follow-up”), and the other 45 participants did not complete follow-up. This study was approved by the Cantonal Ethics Committee Zurich. Written informed consent was obtained from all participants or their legal guardians before enrollment.

**Table 1:** Patient Characteristics at Baseline and Before Matching (N = 108)^*a*^

	Value
Patient demographics	
Age, y	14.83 ± 0.58
Male sex, n	66
Female sex, n	42
Height, m	166.4 ± 7.7
Weight, kg	56.6 ± 9.2
BMI	20.33 ± 2.34
Intersegmental abnormalities	
Signal-reduced disk	17 (18)
High-intensity zone	6 (6)
Disk bulging	12 (13)
Disk herniation	3 (3)
Schmorl nodes	19 (21)
Endplate changes	11 (12)
Pars interarticularis abnormalities	10 (11)
Spondylolysis	2 (2)
Facet joint abnormalities	3 (3)

a Data are given as percentage (n) or mean ± SD unless otherwise indicated. BMI, body mass index; LBP, low back pain.

### Magnetic Resonance Imaging

A routine unenhanced transverse T2-weighted turbo spin echo sequence and a sagittal Dixon T2-weighted sequence were acquired at baseline and at follow-up using a 3-T MRI scanner (MAGNETOM Prisma; Siemens Healthcare). The imaging parameters for baseline and follow-up were equal, with a slice thickness of 4 mm.

### MRI Analysis

MRI scans were assessed for the presence and location of intersegmental abnormalities. These included vertebral disk changes such as signal reduction, disk bulging, disk herniation, annulus fibrosus lesions, and Schmorl herniation (Table 2). Furthermore, structural changes in the vertebral body endplates, facet joints, and interarticular portions were assessed. All MRI scans were analyzed by a fellowship-trained musculoskeletal radiologist (G.C.F.). Analysis was performed using a picture archiving and communication system workstation certified for clinical use (MERLIN 7.1.22; Phönix-PACS GmbH). The images were independently, and in random order, blinded to the clinical information. An analysis of inter- or intrarater agreement was not conducted. However, it has been demonstrated that the assessment of intersegmental abnormalities of the spine generally exhibits substantial to excellent inter- and intrarater agreement.^18^

**Table 2:** Image Analysis of the Lumbar Spine MRI^*a*^

Intersegmental Abnormalities	Description
Signal-reduced disk	Loss of high signal in the nucleus pulposus on sagittal T2-weighted images
High-intensity zone disk tear	Annular fissures characterized by a region of high signal intensity in the otherwise low-signal annulus fibrosus on T2-weighted images^23^
Disk bulging	Displacement of disk material over a segment <25% of the disk circumference; the width of the base is greater than the largest diameter of the disk material protruding beyond the normal disk margins^6^
Disk herniation	Displacement of disk material beyond the edges of the disk space; the diameter of the herniation is greater at the periphery than at the base in the axial or sagittal plane^15^
Schmorl node	Protrusion of disk material through the endplate of the vertebral body and into the adjacent vertebra
Endplate changes	Morphological changes of the vertebral endplates encompassing edema and fatty or sclerotic changes
Pars interarticularis abnormality	Changes such as edema (high T2 signal) or spondylolysis in the pars interarticularis of the vertebral arch
Facet joint abnormalities	Degenerative or inflammatory changes of the facet joint with or without periarticular soft tissue changes

a MRI, magnetic resonance imaging.

### LBP Assessment

To collect and assess information on overuse-related and traumatic injuries as well as general health problems at both baseline and follow-up, we retrospectively interviewed athletes about the 12-month period before the MRI examination. Athletes were classified as symptomatic if at least 1 substantial episode of health problems related to back overuse (defined as those problems affecting training or competition) had occurred in the 12 months before the MRI examination.

### Statistical Analysis

The statistical analysis was performed by 1 author (G.C.F.) using SPSS (Version 28.0; IBM Corp). Descriptive data were reported as the mean with standard deviation and percentage. The prevalence of intersegmental spinal abnormalities was compared between baseline and the 48-month follow-up. Comparisons of the measurements were performed using the Wilcoxon signed-rank test. The Pearson chi-square test was applied to compare the distribution of intersegmental MRI findings between symptomatic and asymptomatic skiers, and the Fisher exact test was used to compare the distribution between groups when the expected count was <5. A significance level of α = .05 was used for all the statistical analyses.

## Results

A total of 63 athletes (mean age at follow-up, 19.6 ± 1.2 years; 25 female) were included in the study. Athletes had a mean body mass index at follow-up of 23.4 ± 0.3, a mean height of 1.73 ± 0.8 m, and a mean weight of 70.3 ± 10.5 kg (Table 3). A significant increase in LBP within the last year affecting training or competition was observed at follow-up compared with baseline (*P* = .04). However, when assessing the overall occurrence of LBP within the last year, no significant difference was detected between baseline and follow-up (*P* = .53). Of the athletes with LBP, 59% (n = 16) reported recurrent LBP, 15% (n = 4) reported permanent back pain, and 26% (n = 7) reported 1-time LBP since baseline.

**Table 3:** Patient Characteristics and Prevalence of LBP at Baseline and 48-Month Follow-up^*a*^

	Baseline (n = 63)	Follow-up (n = 63)	*P* Value^*b*^
Age, y	15.3 ± 1.3	19.6 ± 1.2	
Male sex, n	38	38	
Female sex, n	25	25	
Height, m	1.66 ± 0.2	1.73 ± 0.8	
Weight, kg	57.6 ± 8.8	70.03 ± 10.5	
BMI	20.90 ± 3.1	23.39 ± 2.3	
Back pain within the last year^*c*^	40 (25)	43 (27)	.53
Back pain within the last year affecting training or competition^*c*^	21 (13)	32 (20)	.04
Frequency of LBP in the last 12 mo			
1 time	n/a	26 (7)	
Recurrent	n/a	59 (16)	
Permanent	n/a	15 (4)	

a Data are given as percentage (n) or mean ± SD unless otherwise indicated. BMI, body mass index; LBP, low back pain; n/a, not available. Blank cells indicate that patient characteristics were not compared as they are significantly different in growing children.

b Comparison between baseline and follow-up using the Wilcoxon paired-rank test.

c Assessed via personal interviews on overuse-related injuries.

**Figure 2.**
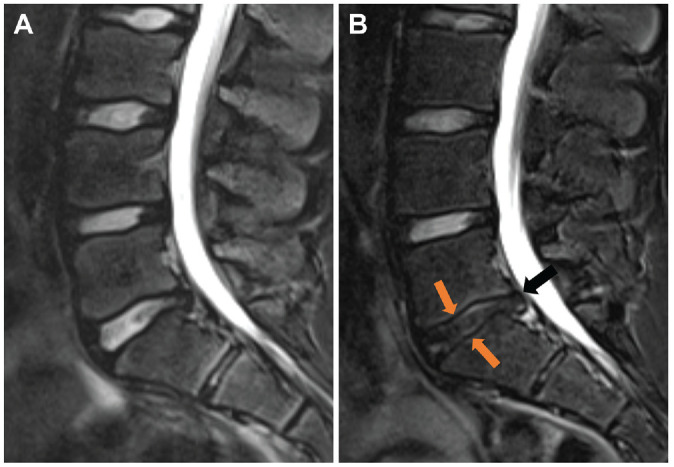
Sagittal Dixon T2-weighted magnetic resonance imaging scans of the lumbar spine of a 19-year-old competitive alpine skier (A) at baseline and (B) after 48 months. Note that in the follow-up image, the excessive progression of disk degeneration resulted in advanced signal reduction (lighter arrows) and progression of disk bulging with a new tear of the annulus fibrosus (black arrow).

**Figure 3.**
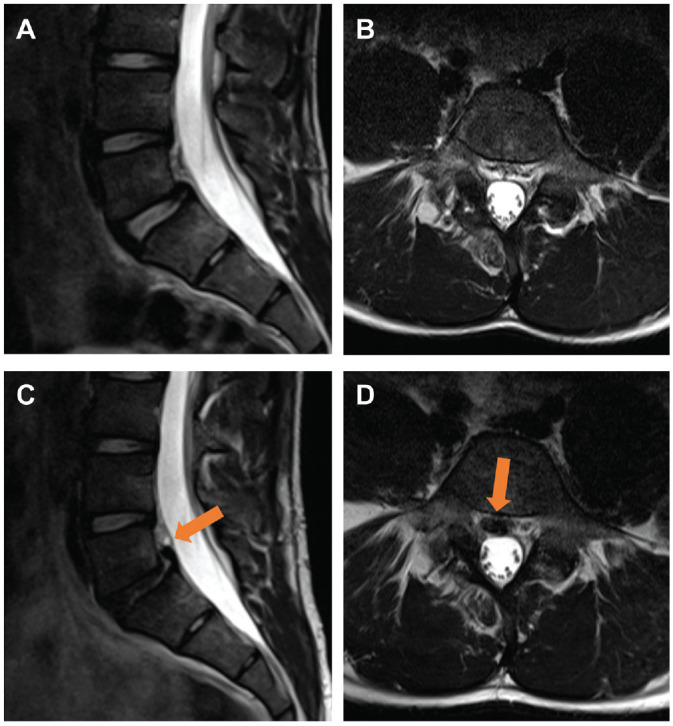
Sagittal Dixon and transverse T2-weighted 3-T magnetic resonance imaging scans of the lumbar spine of an 18-year-old competitive alpine skier (A and B, respectively) at baseline and (C and D, respectively) at the 48-month follow-up. Note that the progression of disk degeneration led to new herniation and the formation of a sequester (arrows), most likely due to the continuous high load applied during alpine skiing. Additionally, anterolisthesis at the L5-S1 level is evident, which is a consequence of the patient's spondylolysis.

**Figure 4.**
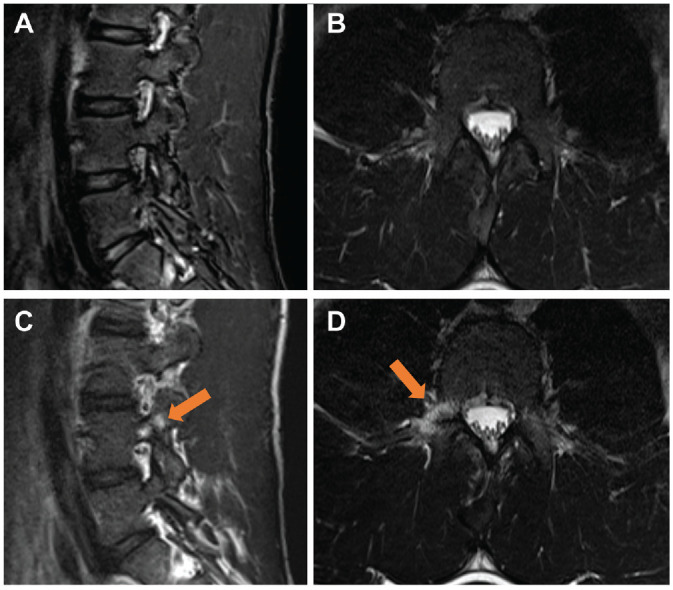
Sagittal Dixon and transverse T2-weighted magnetic resonance imaging scans of the lumbar spine of a 19-year-old competitive alpine skier (A and B, respectively) at baseline and (C and D, respectively) at the 48-month follow-up. Note the bone marrow edema in the right facet joint of the fourth lumbar vertebra (arrows), most likely due to overuse-related changes.

Assessment of intersegmental changes revealed a significant increase in the number of athletes with disk signal reduction (baseline, 16% [n = 10]; follow-up, 33% [n = 21]; *P* = .001) (Table 4), with the L5-S1 segment being the most affected (n = 13) (Figure 2). A significant increase in disk bulging was observed between baseline and follow-up (baseline, n = 7; follow-up, n = 19; *P* = .002), with the L4-5 and L5-S1 segments being most affected (L4-5, n = 10; L5-S1, n = 12). Two new disk herniations were detected at follow-up compared with baseline (*P* = .04) (Figure 3, Table 5). A trend toward an increase in the number of athletes with disk annulus tears was observed, but this increase did not reach statistical significance (*P* = .08). There were no significant changes in the number of patients with abnormalities in the interarticular portion, facet joints, or vertebral endplates (*P* > .05) (Figure 4). Additionally, no improvement or reduction in intersegmental abnormalities was observed in any of the athletes.

**Table 4:** Comparison of Spinal Intersegmental Abnormalities at Baseline and 48-Month Follow-up^*a*^

	Baseline (n = 63)	Follow-up (n = 63)	*P* Value^*b*^
Signal-reduced disk	16 (10)	33 (21)	.001
High-intensity zone	5 (3)	10 (6)	.08
Disk bulging	11 (7)	30 (19)	.002
Disk herniation	2 (1)	5 (3)	.04
Schmorl nodes	21 (13)	16 (10)	.26
Endplate changes	11 (7)	13 (8)	.16
Pars interarticularis abnormalities	6 (4)	6 (4)	>.99
Spondylolysis	3 (2)	3 (2)	>.99
Facet joint abnormalities	2 (1)	5 (3)	.16

a Data are given as percentage (n).

b Pairwise comparison using the Wilcoxon signed-rank test.

**Table 5:** Distribution of Spinal Intersegmental Abnormalities Along the Lumbar Spine at the 48-Month Follow-up^*a*^

	L2-3	L3-4	L4-5	L5-S1
Signal-reduced disk	1	4	6	13
High-intensity zone	0	0	4	2
Disk bulging	7	1	10	12
Disk herniation	0	0	0	3
Endplate changes	2	2	3	3
Pars interarticularis abnormalities	0	0	4	0
Spondylolysis	0	0	0	2
Facet joint abnormalities	0	0	5	0

a Data are given as number of intervertebral segments.

The assessment of intersegmental abnormalities in relation to LBP showed no significant difference between symptomatic and asymptomatic athletes for the overall findings or for each anatomic location at the 48-month follow-up (Table 6). At baseline, athletes with LBP had significantly more signal-reduced disks compared with asymptomatic athletes (*P* = .01). Further assessment of intersegmental abnormalities in relation to activity-limiting LBP showed no significant correlations at follow-up (Table 7). However, at baseline, athletes with activity-limiting LBP had significantly more MRI findings compared with athletes with non–activity-limiting LBP (*P* = .03) (Table 7).

**Table 6:** Comparison of Spinal Intersegmental Abnormalities and Back Pain

	Baseline			48-mo Follow-up		
	Asymptomatic, % (n = 38)	Symptomatic, % (n = 25)	*P* Value^*a*^	Asymptomatic, % (n = 36)	Symptomatic, % (n = 27)	*P* Value^*a*^
≥1 Magnetic resonance imaging finding	68.4	52.0	.19	55.6	55.6	*>*.99
Signal-reduced disk	5.3	28.0	.01	33.3	29.6	.76
High-intensity zone	3.6	8.0	.32	8.3	11.1	.71
Disk bulging	7.9	16.0	.31	27.8	29.6	.87
Disk herniation	0	6.3	.21	2.3	6.9	.79
Schmorl nodes	15.8	28.5	.21	11.1	22.2	.23
Endplate changes	5.3	20.0	.06	8.3	7.4	.89
Pars interarticularis abnormalities	7.9	4.0	.53^*b*^	2.8	11.1	.18^*b*^
Spondylolysis	0	8	.27	0	7.4	.33
Facet joint abnormalities	2.6	0	.41^*b*^	5.6	3.7	.73^*b*^

a Chi-square test unless otherwise indicated.

b Fisher exact test.

**Table 7:** Comparison of Spinal Intersegmental Abnormalities and Substantial, Activity-Limiting Back Pain

	Baseline			48-mo Follow-up		
	Non–Activity Limiting (n = 12)	Activity Limiting (n = 13)	*P* Value^*a*^	Non–Activity Limiting (n = 7)	Activity Limiting (n = 20)	*P* Value^*a*^
≥1 Magnetic resonance imaging finding	25.0	69.2	.03	57.1	55.0	.92
Signal-reduced disk	8.3	46.2	.003	14.3	35.0	.30
High-intensity zone	8.3	7.7	.60	0	15.0	.27
Disk bulging	0	40.8	.03	42.9	25.0	.37
Disk herniation	0	6.3	.73	2.3	6.9	.79
Schmorl nodes	16.7	38.5	.19	10.1	20.2	.23
Endplate changes	8.3	30.8	.04	6.1	8.4	.79
Pars interarticularis abnormalities	8.3	0	.57^*b*^	0	15.0	.28^*b*^
Spondylolysis	8.3	7.7	.60	14	5.0	.29
Facet joint abnormalities	0	0	>.99^*b*^	0	5.0	.55^*b*^

a Chi-square test unless otherwise indicated.

b Fisher exact test.

## Discussion

Overuse-related intersegmental abnormalities of the lumbar spine are common in competitive alpine skiers and are often associated with LBP. This study demonstrated that intersegmental abnormalities, particularly degenerative disk changes, persist throughout skeletal maturation and that some abnormalities even worsen during adolescence. Over a 48-month period, more than twice as many athletes had signal-reduced vertebral disks at follow-up imaging than at baseline. Although the mean age at follow-up was only 19.6 years, 6 athletes already demonstrated a tear of the annulus fibrosus, and 3 athletes exhibited disk herniation. None of the athletes demonstrated an improvement in intersegmental changes between baseline and follow-up.

This progression of intersegmental changes in the lumbar spine of adolescent athletes may be explained by the high loads on the spine that occur during competitive alpine skiing and related off-snow training. In a study by Spörri et al,^20^ the authors analyzed kinematic variables related to disk loading in 8 European Cup–level athletes during giant slalom runs and demonstrated that the spine is exposed to a combined occurrence of frontal bending, lateral bending, and torsion of the trunk. In a similar study, the authors observed increased spinal vibrations in the lower trunk during giant slalom turns at a frequency of 4 to 10 Hz, which is particularly harmful to the spine.^19^ In addition, the accumulation of heavy loads during off-snow training, possibly exacerbated by insufficient recovery time, may lead to overuse-related changes in the spine.^17^ The consistently high exposure of the athletes analyzed in our study to these multiple factors may explain the observed persistent and increasing number of overuse-related injuries of the lower spine.

In our study, disk signal reduction, disk bulging, and disk herniation were most common in the L5-S1 lumbar segment. The intervertebral disk is essentially a fibrocartilaginous joint consisting of a central nucleus pulposus that is rich in proteoglycans and the surrounding annulus fibrosus, a concentric lamellar structure based on type 1 collagen.^10^ Continuous exposure to high loads and stress leads to catabolic processes accompanied by a loss of proteoglycans and, ultimately, to deterioration of the disk and LBP. This was demonstrated by a study that revealed a significant increase in LBP in nonathletic adults with more advanced disk degeneration compared with asymptomatic adults.^5^ Similarly, Peterhans et al^15^ demonstrated that disk degeneration was significantly more prevalent in symptomatic rather than asymptomatic youth competitive alpine skiers, with a mean age of 14.83 years. In our longitudinal study, a significant increase in the number of athletes with LBP affecting training or competition was observed between baseline and follow-up. However, symptomatic athletes did not exhibit significantly more intersegmental abnormalities than asymptomatic athletes at the follow-up MRI. This may be explained by the fact that natural changes in the intervertebral disk, including cell death accompanied by excessive proliferation of chondrocytes and intersegmental changes such as the formation of clefts or radial tears, begin to occur as early as approximately 15 years of age.^2,4^ Therefore, with increasing age, intersegmental changes on MRI scans should be interpreted with more caution and preferably in connection with clinical symptoms. However, the significant increase in overuse-related structural changes detected on follow-up MRI scans highlights the fact that overuse injuries may also remain clinically silent for a longer period of time before causing symptoms at a later stage during the sports career, demonstrating the importance of early screening and proper prevention.

We acknowledge the following limitations of this study. The assessment of intersegmental abnormalities was based solely on MRI morphological data, and no other modality was available for confirmation. A considerable number of participants withdrew from the study between baseline and follow-up examinations (n = 45). The reasons for withdrawal were variable, including termination of professional skiing and loss of interest in the study. The classification of symptomatic and asymptomatic skiers was based on retrospective interviews relating to the 12-month period before the MRI examination, which may have led to some recall bias, particularly for less severe and short/single episodes of pain. However, competitive alpine skiers may have been better able to recall severe or recurrent symptoms with certainty. Moreover, the number and severity of potential previous injuries experienced by the athletes throughout their careers were not evaluated. Accordingly, the potential for confounding effects of cumulative trauma could not be investigated.

## Conclusion

Overuse-related intersegmental abnormalities of the lumbar spine are common in competitive alpine skiers and may persist throughout skeletal maturation and even worsen during adolescence. Given the high prevalence of competitive athletes with LBP, considering these factors is important for prevention and treatment.
